# Spatiotemporal Characteristics of Air Quality across Weifang from 2014–2018

**DOI:** 10.3390/ijerph16173122

**Published:** 2019-08-27

**Authors:** Chengming Li, Zhaoxin Dai, Lina Yang, Zhaoting Ma

**Affiliations:** Chinese Academy of Surveying and Mapping, Beijing 100830, China

**Keywords:** air quality index, primary pollutants, temporal characteristics, spatial characteristics, Weifang

## Abstract

Air pollution has become a severe threat and challenge in China. Focusing on air quality in a heavily polluted city (Weifang Cty), this study aims to investigate spatial and temporal distribution characteristics of air pollution and identify the influence of weather factors on primary pollutants in Weifang over a long period from 2014–2018. The results indicate the annual Air quality Index (AQI) in Weifang has decreased since 2014 but is still far from the standard for excellent air quality. The primary pollutants are O_3_ (Ozone), PM_10_ (Particles with aerodynamic diameter ≤10 µm), and PM_2.5_ (Particles with aerodynamic diameter ≤10 µm); the annual concentrations of PM_10_ and PM_2.5_ show a significant reduction but that of O_3_ is basically unchanged. Seasonally, PM_10_ and PM_2.5_ show a U-shaped pattern, while O_3_ exhibits inverted U-shaped variations, and different pollutants also present different characteristics daily. Spatially, O_3_ exhibits a high level in the central region and a low level in the rural areas, while PM_10_ and PM_2.5_ are high in the northwest and low in the southeast. Additionally, the concentration of pollutants is greatly affected by meteorological factors, with PM_2.5_ being negatively correlated with temperature and wind speed, while O_3_ is positively correlated with the temperature. This research investigated the spatiotemporal characteristics of the air pollution and provided important policy advice based on the findings, which can be used to mitigate air pollution.

## 1. Introduction

Since the Chinese economic reform, with the rapid industrial development and growing energy consumption in China, air pollutant emissions have increased continuously, thus exacerbating air pollution. For example, in January 2013, a wide range and long duration of hazy weather occurred in Central and eastern China, raising public concern about urban air quality [1,2,3]. To address more serious air pollution, the Chinese government announced the new National Ambient Air Quality Standards in 2012 and completed all-round monitoring of air quality in cities at the prefecture level in 2015. By 2016, air quality monitoring was implemented in 338 cities in China. Air pollution can cause respiratory, cardiovascular, and cerebrovascular diseases through gas exchange in the lungs [4,5]. Air pollution also reduces atmospheric visibility and causes smog [6,7] and inconvenience to travelers. Therefore, it is critical to accurately identify the temporal and spatial characteristics of air quality and its main influential factors and to correspondingly control and alleviate air pollution.

At present, existing research on the temporal and spatial characteristics of air quality can be classified into three types: Studies based on remote sensing inversion of the aerosol optical depth (AOD), studies based on ground monitoring stations and studies based on modelling which is complementary to satellite and ground-based observations; Studies based on remote sensing inversion usually focus on large-scale areas to provide a regional overview [8]. Due to the high frequency monitoring, research based on real-time ground-monitored data is significant to better explore the detailed patterns (seasonal, and diurnal) especially at the city scale, for instance, for medium- and small-scale areas. This study focuses on the analysis based on ground-monitored data.

Many previous studies focused on the spatiotemporal characteristic analysis of air pollution in large-scale regions. For example, Zhan et al. [9] analyzed the AQI in 338 cities in China and revealed that air pollution in China was still serious in 2015, with PM_2.5_, PM_10_, and O_3_ being the primary pollutants, and the temporal and spatial characteristics were observed to differ in various cities. Using ground monitoring data, Zhao et al. [10] analyzed the temporal and spatial variations in PM_2.5_, PM_10_, SO_2_, and NO_2_ in five areas, namely, the Yangtze River Basin, the Bohai Economic Rim, the Pearl River Delta, the Central Urban Agglomeration, and the Western Urban Agglomeration, and the results indicated that since 2013, the concentrations of most pollutants decreased. Yan et al. [11] conducted statistical analyses on the PM_2.5_ concentrations in 13 cities in the Beijing-Tianjin-Hebei region in 2016 and found that the PM_2.5_ concentration was the highest in Winter (December–February) but the lowest in Summer (June to August). Spatially, the PM_2.5_ concentration was higher in cities far away from the Bohai Bay, but lower in coastal cities. Hu et al. [12] analyzed the temporal and spatial characteristics of the PM_2.5_ and PM_10_ concentration distributions in 33 cities in the North China Plain and the Yangtze River Delta based on hourly particulate matter (PM) concentration data from January–August 2013. The results suggested that the PM_2.5_ concentrations in these two regions exceed the standard of the World Health Organization (WHO), and higher proportions of coarse-grained particles are found in these regions [12].

With respect to studies on small analytical units, based on nine national monitoring stations, Chen et al. [13] discovered that the PM_2.5_ concentration in Nanjing varies significantly seasonally, and the PM_2.5_ concentration is the highest in Winter but the lowest in Summer, while the peak concentration is higher at night than that during the day. Based on research on 10 national PM monitoring stations in Gansu in 2014, Guan et al. [14] pointed out that the PM_10_ concentration is the highest in March in Gansu because of frequent springtime sandstorms, while the PM_2.5_ concentration reaches its peak in January due to home heating. Zhao et al. [15] reported that cities in the southwestern part of the Sichuan Basin have higher change rates of the PM_2.5_ concentration compared to other cities by analyzing the daily PM_2.5_ concentrations from 22 ground monitoring stations, and the researchers indicated that the PM_2.5_ concentration is negatively correlated with the boundary layer height and wind speed but positively correlated with the air temperature. Xu et al. [16] showed that the annual average PM_2.5_/PM_10_ ratios in urban areas have distinct seasonal, monthly, and daily variation patterns; the ratios increase significantly along the spatial gradient from the rural areas and the boundaries of the urban areas to the core regions of the cities.

However, due to the incomplete ground monitoring data and the complication of processing of a very large amount of long-term data, there are limitations in the state-of-the-art similar studies. First, most studies focused on typical and large-scale regions, like Beijing-Tianjin-Hebei [17,18] and the Yangtze River Delta [19], with little research paid attention to small-scale areas, especially in heavily polluted Weifang City [20]. Furthermore, studies mostly focused on only a single pollutant, such as PM_2.5_ or PM_10_, but people are seldomly exposed to a single pollutant [12], and analysis of various pollutants is more valuable. Third, studies mostly used approximate and historical data from nationally controlled monitoring stations (fewer than 20 in number when focusing on one city), leading to low spatial resolutions; moreover, the existing studies mostly used daily data during short periods (such as one year), resulting in low temporal resolution.

These limitations motivate this study. This paper uses hourly high-frequency ground monitoring data from 38 national and provincial monitoring stations in a heavily polluted city, Weifang, from 2014–2018, and adopts efficient statistical methods, such as frequent pattern mining, to comprehensively explore the temporal and spatial characteristics of the AQI and the primary pollutants in the city. The objectives of this paper are: (1) to analyze for the first time the characteristics of AQI during a long period from 2014–2018 in Weifang City; (2) to explore the spatial-temporal patterns of primary pollutants influencing the AQI in Weifang City; and (3) to assess the correlations between primary pollutants and meteorological factors quantitatively. This paper helps to improve the understanding of the temporal and spatial characteristics and the mechanisms of air pollution in Weifang City, and can provide scientific and technical support for subsequent air pollution research. Furthermore, the study helps to formulate effective prevention and control measures and offers policy advice specifically for environmental management in Weifang and other similar areas.

This paper includes four sections. Section 2 describes data sources and methodologies. The temporal and spatial characteristics and the influential factors of the air quality in Weifang are explained in Section 3, followed by the conclusions in Section 4.

## 2. Data and Methods

### 2.1. Study Area and Data

#### 2.1.1. Study Area

**Figure 1:**
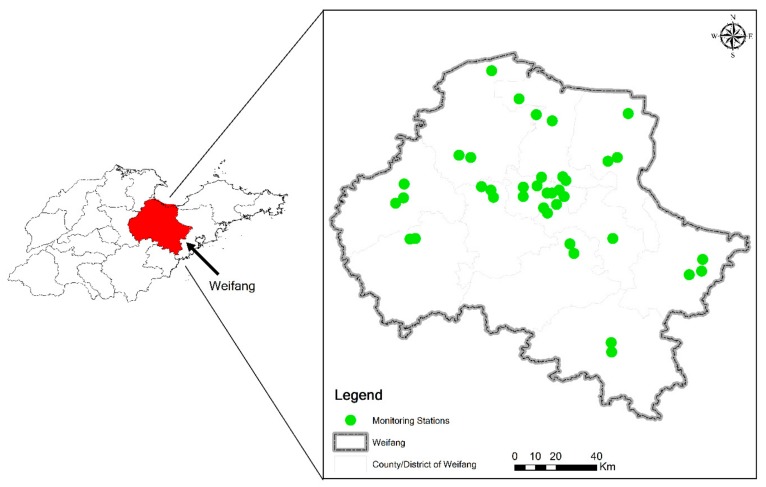
Study area and spatial distribution of the 38 monitoring stations in Weifang.

Weifang City is located in the middle of the Shandong Peninsula (Figure 1), with Zibo City to the west, Linyi City to the south, Qingdao to the east and Laizhou Bay, Bohai Sea, to the north, between 118.17° E–120.01° E and 35.68° N–37.43° N. The study area includes four districts, six cities and two counties, with a total area of 16,000 km^2^. Weifang City is high in the south and low in the north. The southern part is dominated by low hills, while the northeastern part consists of mainly plains, bays and rivers. Weifang is a semihumid area with a temperate continental monsoon climate. In 2018, Weifang was one of the most rapidly developing cities in Shandong Province. There have been rapid advancements in various fields, such as industry, transportation, and economy. However, the air pollution that comes with these developments has become increasingly serious. The air quality of the city is slightly below average compared to both those of other cities in Shandong and those of key cities in China. Nevertheless, there are few studies and reports on different air pollution indicators in Weifang. Investigation and analysis of the temporal and spatial characteristics of air pollutants are therefore important for improving the air quality in Weifang.

#### 2.1.2. Data Sources

The monitoring station data used in this study were obtained from 5 national stations, 4 provincial stations, and 29 urban stations in Weifang and included six air pollutants, namely, coarse particulate matter (PM_10_), fine particulate matter (PM_2.5_), ozone (O_3_), nitrogen dioxide (NO_2_), sulfur dioxide (SO_2_), and carbon monoxide (CO). Data are retrieved by the monitors every five minutes. All the data used in this paper are in units of hours, which are calculated as the average of the measurements taken every five minutes. The meteorological data used in this paper are from the Weifang Statistical Bulletin, including the wind speed and air temperature.

The AQI is a dimensionless index that quantitatively describes the air quality. According to the new National Ambient Air Quality Standards (GB 3095-2012) implemented by the Ministry of Environmental Protection (now known as the Ministry of Ecology and Environment) of the People’s Republic of China in 2012, different individual air quality indices (IAQIs) of various factors (PM_10_, PM_2.5_, O_3_, NO_2_, SO_2_, and CO) are first computed, and then the highest IAQI value is taken as the value of the AQI. The equation is given as follows.

(1) $$IAQI_{p=}\frac{IAQI_{Hi}-IAQI_{Lo}}{C_{Hi}-C_{Lo}}\left(C_{p}-C_{Lo}\right)+IAQI_{Lo}$$

(2) $$AQI=\max\left(IAQI_{1},IAQI_{2},IAQI_{3},\ldots ,IAQI_{n}\right)$$

When AQI > 50, the factor with the highest IAQI represents the major pollutant. There are six AQI categories, and their ranges are 0–50, 51–100, 101–150, 151–200, 201–300, and >300. The corresponding pollution levels are excellent, good, lightly polluted, moderately polluted, heavily polluted, and severely polluted, respectively. The higher the AQI, the worse the air quality becomes [21].

### 2.2. Data Processing

Up to 10 million air quality data points are used in this paper. Traditional Geographical Information System (GIS)-based statistical analysis methods cannot satisfy the processing of a large amount of data. To achieve efficient calculations and analyses and to allow interactive visualization of the temporal and spatial characteristics of the air pollutants, the frequent pattern (FP)-growth algorithm is used in this paper [22].

The FP-growth algorithm is an FP mining algorithm based on the FP-tree structure. FP-tree structure is an extended prefix-tree structure for storing compressed, crucial information about frequent patterns. The basic idea of this algorithm is as follows. First, for each item, its conditional projection database and projection FP-tree are constructed. Then, this process is repeated for each newly built FP-tree until the constructed new FP-tree is empty or contains only one path. Finally, when the constructed FP-tree is empty, its prefix is frequent pattern.

The FP-growth algorithm can improve efficiency of mining with techniques as follows. By preserving key information of each set, a large database in the transaction database is compressed into an FP-tree, which can avoid costly, repeated database scanning. Furthermore, a partitioning-based method is used to decompose the mining task into a set of smaller tasks for mining confined patterns in conditional databases, which dramatically reduces the search space [22]. The FP-growth algorithm utilizes tree structures to compress transactions, which greatly enhances the efficiency for a large amount of data processing [23]. Han et al. reported that the FP-growth method is efficient and scalable for mining both long and short frequent patterns, and is about an order of magnitude faster than the Apriori algorithm [22]. This paper employs the FP-growth algorithm based on the Spark platform.

A valid check on hourly data was conducted to eliminate problematic and missing data points before data processing tasks. Statistical analysis and ArcGIS are used to explore the temporal and spatial patterns of air pollution. For temporal statistical analysis, C++ language is applied in this study to obtain annually, seasonal, monthly, and daily variations of air pollutants; these variations are calculated by the arithmetic means of the hourly renewed data from 38 ground monitoring stations from 2014–2018. For spatial analysis, spatial characterization is conducted through the use of spatial analysis methods with the support of ArcGIS software (published by the Environmental Systems Research Institute (ESRI), Redlands, California, USA).

## 3. Results and Discussion

### 3.1. Urban Air Quality in Weifang

#### 3.1.1. AQI Characteristics

**Figure 2:**
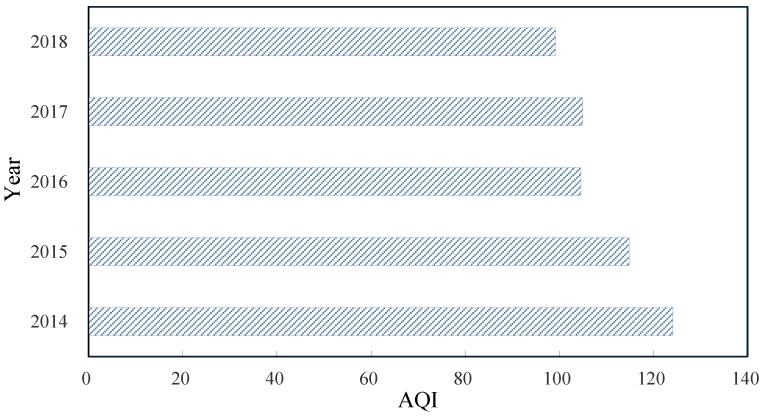
Annual average of the AQI in Weifang from 2014–2018.

Figure 2 shows the variation characteristics of the annual average AQI in Weifang from 2014–2018. The figure indicates that the AQI in Weifang is high. From 2014–2017, the AQI is higher than 100, which is slightly polluted. Specifically, the air pollution in Weifang is serious in 2014, with an average AQI of 124. In subsequent years, the value decreases, with a decrease of 20.16%. However, the value is still far from the standard for excellent air quality, where AQI < 50. The decreasing trend may be closely related to the environmental protection actions taken under the eight major initiatives, including controls on pollution from industry, fossil fuel consumption, and motor oil and gas consumption in 2017.

**Figure 3:**
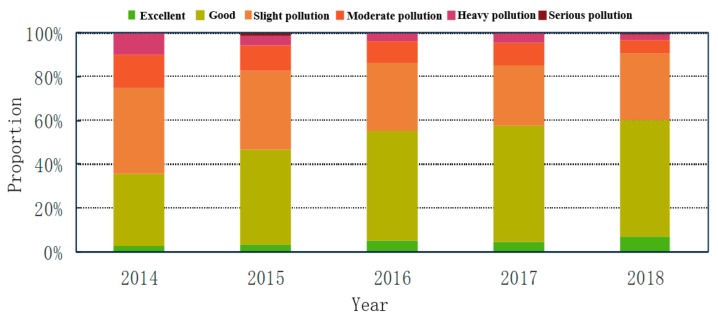
Statistical analysis of the different AQI categories from 2014–2018 in Weifang.

Figure 3 illustrates the proportions of days with different AQI categories in Weifang from 2014–2018. In the past five years, the proportion of days with excellent and good air quality to the total number of days has gradually increased. The proportion reaches 60% in 2018, but there is still a gap between this value and the expected value of 80% of the Chinese government. The number of days when the city is slightly polluted decreases gradually from 2014–2017, but the number of slightly polluted days rises slightly in 2018. The number of days when the city is heavily or severely polluted significantly decreased by 20% in 2018 compared to 2014. However, there are still days when the city is severely polluted.

**Figure 4:**
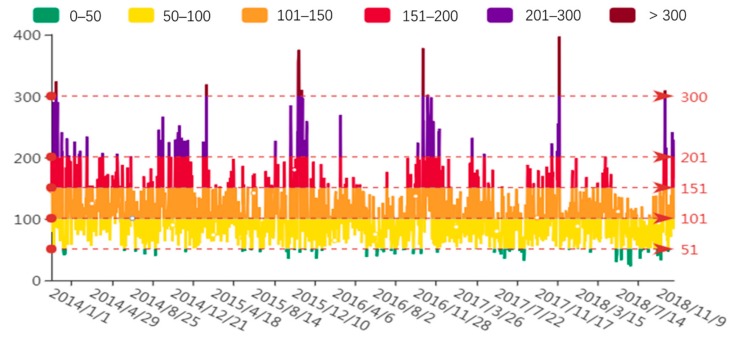
Variations in the daily average AQI in Weifang from 2014–2018.

The variations in the daily average AQI in Weifang from 2014–2018 are shown in Figure 4. There are certain periodic characteristics, with five U-shaped distributions. From 2014–2018, the number of days when the AQI falls in the second category (good air quality) is the greatest, accounting for approximately 49% of the total days. Days with good air quality (51 < AQI < 100) are often found in July, August, and September. This phenomenon is because pollutant diffusion and dilution are more favorable when precipitation and vegetation coverage increase, and surface convection becomes stronger in Summer. The number of days when the AQI falls in the slightly polluted level ranks second (29%). The number of days with moderate pollution accounts for approximately 12%, while those with AQI > 200 (heavy and severe pollution) account for approximately 5% of the total days. The days with heavy and severe pollution occur mostly in January, November, and December, which is consistent with many other studies [9,24]. This finding may be because the meteorological conditions in Winter, such as frequent temperature inversion, low air temperatures, weak convection, little precipitation, and low humidity, do not facilitate air movement in Weifang. Furthermore, heating during Winter leads to a surge in fossil fuel consumption and thus increased emissions of pollutants [25]. In summary, the AQI in Weifang is high in Winter and low in Summer.

#### 3.1.2. Primary Pollutants Affecting the AQI in Weifang

**Figure 5:**
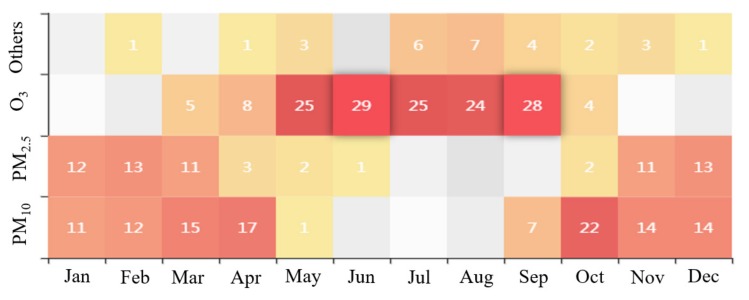
Frequency of the different pollutants being the primary pollutant in the different months.

Statistical analyses are conducted to calculate the proportions of days when different pollutants become the major pollutants in Weifang (Figure 5) in 2018. The results demonstrate that the number of days when O_3_ is the major pollutant in the city is the greatest (148 days) and accounts for approximately 40.55% of the total days, followed by PM_10_ and PM_2.5_, with percentages of 31.25% and 20.82%, respectively. However, the days when NO_2_, SO_2_, and CO are the major pollutants account for only 7.67% of the total days. This finding is similar to the conclusions of Zhan et al. [9] and Song et al. [26], who performed AQI analyses across China and discovered that PM_2.5_, PM_10_, and O_3_ are the major air pollutants. However, the proportion of days when PM_2.5_ is the major pollutant is the highest for mainland China and that of O_3_ is the highest in Weifang [26].

To more specifically analyze the mechanisms affecting the air quality in Weifang, this paper evaluates the temporal and spatial characteristics of the top 3 primary pollutant (O_3_, PM_10_, and PM_2.5_) that affect the AQI separately.

### 3.2. Temporal Characteristics of the Primary Pollutants

#### 3.2.1. Annual Characteristics

**Figure 6:**
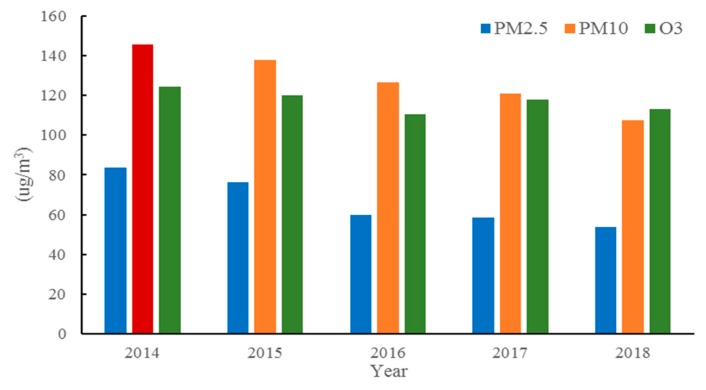
Annual average concentration of the pollutants that affect the AQI in Weifang.

Figure 6 shows the annual average concentrations of the six pollutants in Weifang from 2014–2018. The annual average PM_10_ concentration in 2018 is 107.3 μg/m^3^, which is 38.22 μg/m^3^ lower than that in 2014, with a percentage decrease of 26.3%. In addition, compared to that in 2014, the annual average PM_2.5_ concentration in 2018 decreased by 29.59 μg/m^3^ (35.4%) and reached 53.99 μg/m^3^. Nevertheless, the annual average PM_2.5_ concentration still exceeded the grade II standard of the Chinese Ambient Air Quality Standards (CAAQS) (35 µg/m^3^). Unlike the PM concentration cases, the concentrations of O_3_ always remain at high levels from 2014–2018 and exceed the national first-level standard (100 μg/m^3^). Unlike haze, O_3_ pollution is not visible, resulting in neglect. However, excessive intake of O_3_ can cause lung failure. The government and citizens should pay more attention to O_3_ pollution and take effective measures to alleviate O_3_ pollution [26,27].

#### 3.2.2. Seasonal and Monthly Characteristics

**Figure 7:**
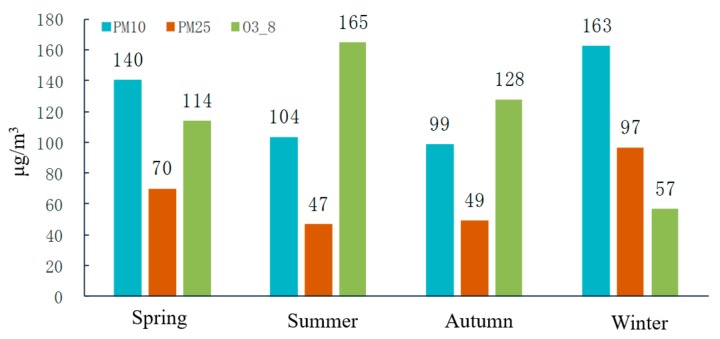
Seasonal concentration characteristics of the primary pollutants.

**Figure 8:**
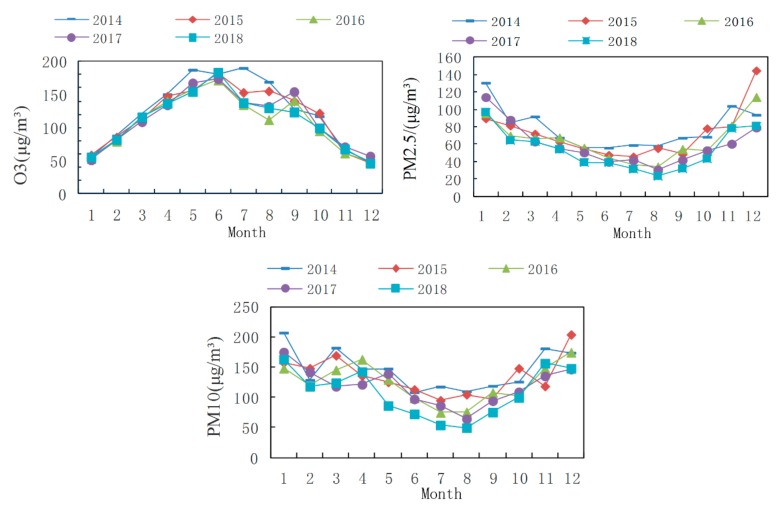
Monthly characteristics of the primary pollutants from 2014–2018.

The statistical seasonal and monthly characteristics of the primary pollutants in Weifang are illustrated in Figure 7 and Figure 8, respectively. In Winter, PM_2.5_ and PM_10_ are the primary pollutants. In Summer, O_3_ becomes the major pollutant, followed by PM_10_. Specifically, the annual peak of the daily maximum 8-hour concentration of O_3_ is found in summer, and the maximum concentration occurs in June. An annual trough appears in Winter, and the minimum concentration is reached in January and December. This phenomenon results in a distinct inverted U-shaped variation curve with seasonal O_3_ concentrations in the ascending order of winter > spring > autumn > summer. In contrast to the monthly O_3_ variations, the variations in the PM_2.5_ concentrations follow a distinct U-shaped curve. A trough appears in summer, which agrees well with previous studies [28,29]. The minimum concentration normally occurs in August, whereas the maximum is reached in January and December. The seasonal PM_2.5_ concentrations follow the ascending order of summer > autumn > spring > winter. The PM_10_ variations are similar to those in PM_2.5_, but the PM_10_ trough occurs in autumn, and the seasonal PM_10_ concentrations can be arranged in the ascending order of autumn > summer > spring > winter.

The O_3_ concentration in Weifang increases in summer, probably because intense sunshine, prolonged solar radiation, and high temperatures during summer are conducive to more intense photosynthesis, thereby promoting the conversion of nitrogen oxides and volatile organic pollutants into O_3_ [26,30]. The O_3_ concentration is significantly and positively correlated with the air temperature. In contrast, the high regional temperatures, stronger solar radiation, rapid surface convection, and increased precipitation and vegetation coverage in Summer facilitate air movement and thus promote diffusion and dilution of atmospheric PM, such as PM_2.5_ and PM_10_ [31]. In addition, weak regional convection and a relatively stable atmosphere in Winter easily result in inversion layers, and this situation is not conducive to the diffusion of atmospheric PM. Moreover, both central heating and greater energy consumption in Winter give rise to a higher PM_2.5_ concentration.

#### 3.2.3. Daily Characteristics

**Figure 9:**
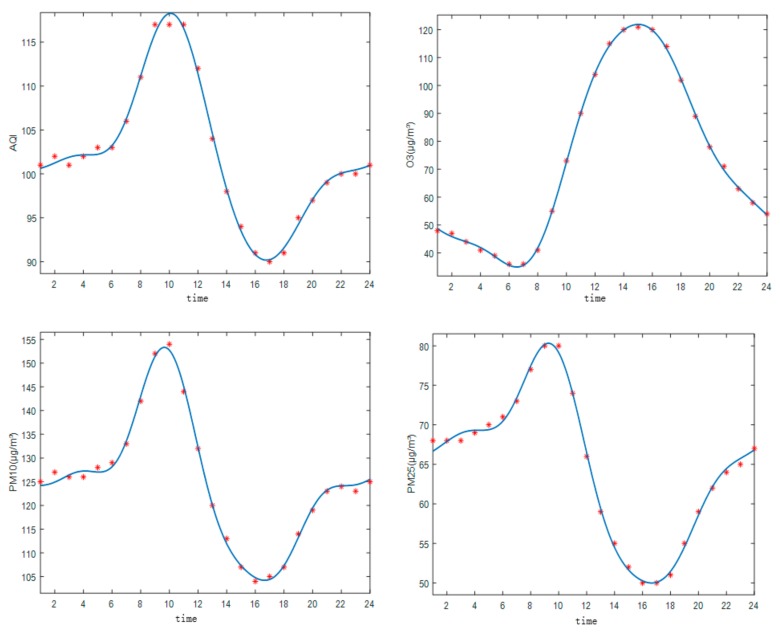
Fitting curves of the AQI and major pollutant concentration.

The Fourier fitting method is used to conduct Fourier approximation analysis of the characteristics of the daily average concentrations of the primary pollutants in Weifang from 2014–2018. The Fourier approximation functions of the AQI and the primary pollutants, O_3_, PM_10_, and PM_2.5_, are shown in Figure 9 (multiple determination coefficient R2 > 0.99).

As illustrated in Figure 9, the concentrations of both PM_10_ and PM_2.5_ peak at approximately 09:00. The peak concentrations are 153 μg/m^3^ and 82 μg/m^3^, respectively. The troughs are observed at approximately 16:00, probably because human activities become less intense at this time, leading to a reduction in atmospheric PM emissions. Furthermore, there is sufficient time for most pollutants to gradually diffuse. This finding is partly in agreement with previous studies of Wang et al. [32], who showed that the daily average value of PM_2.5_ concentration peaked at 10:00 and the valley occurred at 16:00. In contrast, the characteristics of O_3_ are opposite to those of the AQI, PM_10_, and PM_2.5_. The minimum O_3_ concentration of 40 μg/m^3^ is observed from 06:00–07:00; then, the concentration increases rapidly and reaches its maximum of 123 μg/m^3^ from 14:00–16:00, suggesting that the O_3_ concentration is positively correlated with the solar radiation intensity. In general, the daily characteristics of PM_2.5_, PM_10_, and AQI are greatly similar, but the AQI shows some delay compared to PM_2.5_ and PM_10_.

### 3.3. Spatial Characteristics of the Primary Pollutants that Affect the AQI

**Figure 10:**
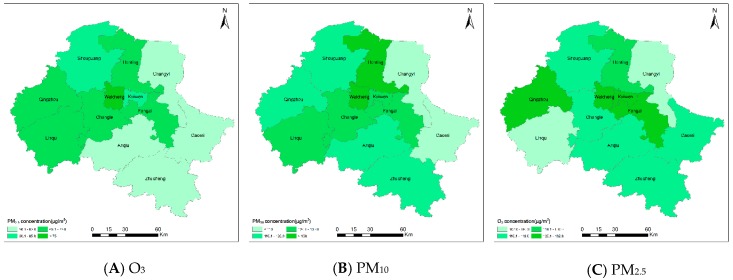
Spatial distribution of the primary pollutant concentrations in Weifang. (**A**) O_3_ concentration; (**B**) PM_10_ concentration; (**C**) PM_2.5_ concentration.

The spatial distribution of the annual average concentrations of the primary pollutants is shown in Figure 10. One should note that the county/district air pollution level is represented by the average of points where concentrations are monitored within the area, which may lead to extrapolation errors/bias. However, generally speaking, these statistical results provide a way to understand the spatial distribution characteristics of regional air pollution, which support the local government decision-making for environmental governance.

In general, the O_3_ concentration is high in the central region of the city and low in the rural areas. In particular, the concentrations in both Weicheng District and Kuiwen District exceed 130 μg/m^3^. The reason may be that the O_3_ concentration is greatly influenced by the intensive human activities in the central region, resulting in significant ozone pollution effects. According to the daily characteristics of the O_3_ concentration, the government can focus on the central region and take proper measures to strictly control volatile organic pollutant and vehicle emissions during the period when the O_3_ concentration peaks (approximately 15:00) [33].

The PM_10_ concentration presents a west-high, east-low trend and lies between 90 μg/m^3^ and 140 μg/m^3^. The concentrations in the west basically exceed 120 μg/m^3^, while those in the southeast are normally approximately 110 μg/m^3^. In particular, the highest concentration of 135.14 μg/m^3^ is observed in the Weicheng District. The concentrations in the Hanting District and Linqu are 131.02 μg/m^3^ and 129.33 μg/m^3^, respectively. Gaomi and Changyi have the lowest concentrations of 103.30 μg/m^3^ and 106.75 μg/m^3^, respectively.

The PM_2.5_ concentration in Weifang shares similar spatial characteristics with the PM_10_ concentration, with higher values in the west compared to the southwest. The regional average concentration falls within the range of 55–85 μg/m^3^. Specifically, the highest PM_2.5_ concentration of 83.38 μg/m^3^ is measured in the Weicheng District, followed by that in Qingzhou (74.22 μg/m^3^). Anqiu and Changyi have the lowest concentrations of 58.24 μg/m3 and 56.13 μg/m^3^, respectively.

There may be two reasons for the spatial characteristics of PM_2.5_ and PM_10_. One reason is the topography of the region: the southern part of Weifang consists mostly of low hills with extensive vegetation, which can effectively adsorb atmospheric particulates and reduce the amount of ground aerosols reaching the atmosphere, which thereby lowers the PM concentrations [20]; in contrast, the western Weifang area includes Shouguang and Qingzhou counties, and is adjacent to Zibo City, a heavy industrial city with more severe air pollution. Substantial air pollutants and toxic particles from Zibo City can be transported to the western areas, resulting in an intense air pollution effect. The other reason is related to the rapid population growth in the western cities, such as Linqu and Qingzhou. The population densities in these cities are higher, resulting in increased residential emissions of atmospheric pollutants and therefore higher regional PM concentrations [34].

### 3.4. Correlation Between the Primary Pollutants and Meteorological Factors

The above analysis shows that PM_10_ and PM_2.5_ have similar characteristics, so in this section, the correlations among PM_2.5_, which represents PM_10,_ and O_3_ and meteorological factors are analyzed.

**Figure 11:**
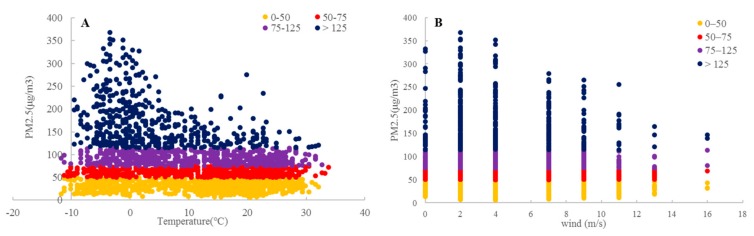
Correlations between the PM_2.5_ concentration and meteorological factors. (**A**) temperature; (**B**) wind speed.

Figure 11 shows the correlations of the PM_2.5_ concentration with meteorological factors. As illustrated in panel A, the PM_2.5_ concentration has a negative relationship with the temperature. The PM_2.5_ concentration is higher from −6–(−5 ℃), when PM pollution occurs frequently. It can be deduced that this temperature range is the most conducive to PM_2.5_ generation. In addition, this temperature range is not conducive to pollutant diffusion, easily leading to accumulation. When the temperature exceeds 15 ℃, the PM_2.5_ concentration decreases, possibly because high temperatures and abundant precipitation in Summer help to effectively wash away pollutants. As shown in panel B, the PM_2.5_ concentration also has a negative relationship with the wind speed. More specifically, when the wind speed is 0–4 m/s, the frequency of serious PM_2.5_ pollution is the highest. However, when the wind speed is higher than 4 m/s, the severe pollution cases decrease, suggesting that at such wind speeds, winds facilitate diffusion and dilution of atmospheric pollutants more effectively, while winds slower than 4 m/s can weaken these effects. In addition, winds slower than 4 m/s will entrain dust, leading to increased pollutant concentrations in certain localities. Hence, at wind speeds of 0–2 m/s, effective measures should be taken in advance to avoid exacerbated air pollution due to PM diffusion. These findings echo many previous studies which showed a negative correlation between the temperature, wind and the air pollution [2]. However, the same finding was not observed in the study of Zhan et al. [9], who presented a positive relationship between AQI and wind speed and reported that both downwind areas and dust storm-prone areas are likely to suffer serious air pollution by increasing wind speed.

**Figure 12:**
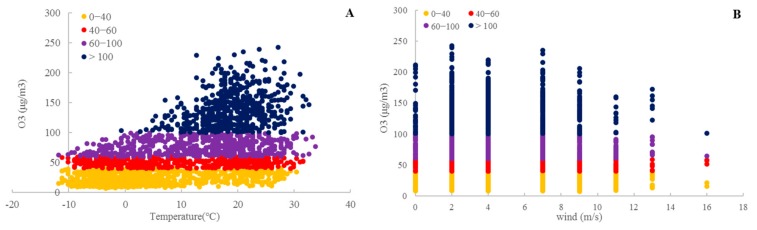
Correlations between the O_3_ concentration and meteorological factors. (**A**) temperature; (**B**) wind speed.

Figure 12 shows the correlations of the O_3_ concentration with meteorological factors. In contrast to PM_2.5_, the O_3_ concentration has a positive relationship with the temperature. The O_3_ concentration is higher at approximately 25 ℃. The higher the temperature, the higher the O_3_ concentration. Similar to PM_2.5_, the O_3_ concentration has a negative relationship with the wind speed. When the wind speed exceeds 8 m/s, O_3_ pollution decreases. Compared with the correlation between the PM_2.5_ concentration and wind speed, the relationship between the O_3_ concentration and wind speed is not significant. The findings in this study can support the results of some previous studies in the terms of quantitative analysis.

### 3.5. Policy Implications

Some effective policy advice based on the findings of this study is presented to reduce air pollution. First, the main primary pollutants influencing AQI in Weifang are O_3_, PM_10_ and PM_2.5_, which should receive more attention by the government. According to their spatio-temporal characteristics, more emphasis should be placed on mitigating PM_10_ and PM_2.5_ in winter and in western regions, and O_3_ pollution in Summer, which is mainly distributed in central areas.

Second, O_3_ is the primary pollutant influencing AQI in Weifang, which is not a special case. Studies in Shenzhen [33] and Beijing [27] also showed similar conclusions. With more efforts were devoted to control the pollution of particulate matter, such as PM_2.5_, near-surface ozone will be another key factor affecting China’s air quality after particulate matter. However, the attention to ozone in China is still in its infancy. The Chinese government should pay more attention to ozone pollution. For instance, the O_3_ concentration is closely related to intense human activities and temperature, which are usually high in Summer and at approximately 15:00 daily, and initiatives such as strictly controlling volatile organic pollutants and vehicle emissions should be implemented by the government in the central region during the period when O_3_ concentration peaks (15:00 pm).

Third, serious air pollution (such as PM_10_ and PM_2.5_) occurred in western Weifang (such as Linqu and Qingzhou) due to two reasons. One is related to the local high population density, the other is correlated with the air pollution transportation from adjacent areas. Zibo City is located on the western side of Weifang City, a heavy industrial city with more severe air pollution. The air pollutants from Zibo City can be transported to the western areas in Weifang. The government should promote the improvement of industrial structure, implement and set pollutant emission standards and use clean energy to decrease air pollution. Additionally, this result also implies that it is a global problem for air pollution mitigation, and implying that is significant for regional cooperation [35]. When formulating measures to prevent and control air pollution, attention should be paid to the interaction between cities and surrounding areas, and the joint prevention and cooperation between regions should be strengthened.

Finally, vegetation plays an important role in air quality. Government can plant more trees in heavily polluted areas, which can significantly increase the surface leaf area and relative humidity, achieve pollutant adsorption and a dilution effect, and effectively reduce air pollution [24].

## 4. Conclusions

Using hourly ground-monitoring data from 2014–2018 in Weifang City, this study examined the temporal and spatial characteristics of the AQI in Weifang along with its primary pollutants (O_3_, PM_10_ and PM_2.5_) and analyzed the relationships between the major pollutants of the city and meteorological factors. The major conclusions are as follows.

(a) The results showed that the AQI in Weifang was higher than 100 from 2014–2017, and decreased significantly since 2017. In the last five years, the annual average proportion of days with excellent and good air quality has increased gradually, reaching 60% in 2018, while the number of days with heavy or severe pollution has decreased substantially (declining by 20%). This may be because environmental protection actions were implemented under the eight major initiatives in 2017, which are beneficial for air pollution mitigation.
(b) The days when AQI > 200 (heavy and severe pollution) are mostly concentrated in January, November, and December. Overall, the AQI is high in Winter and low in Summer. This finding is related to the meteorological conditions and heat-supply during winters.
(c) The primary pollutants in Weifang are O_3_, PM_10_, and PM_2.5_, accounting for 40.55%, 31.23%, and 20.82%, respectively, of the AQI. From 2014–2018, both PM_2.5_ and PM_10_ pollution levels significantly decreased, whereas O_3_ remained basically unchanged. The seasonal and monthly variations in the PM_10_ and PM_2.5_ concentrations show U-shaped curves with highs in Winter and at approximately 09:00 daily, while O_3_ follows an inverted U-shaped curve and is high in Summer at approximately 15:00 daily. This finding infers that near-surface ozone will become another key factor influencing air quality after particulate matter, and more attention should be paid to ozone pollution. The high level of O_3_ pollution in Summer may be associated with the intense sunshine and prolonged solar radiation. In Summer, initiatives such as strictly controlling volatile organic pollutant and vehicle emissions should be implemented during the period when O_3_ concentration peaks (15:00 pm).
(d) Spatially, there is a high O_3_ pollution level in the central region but a low level in the rural areas, while the PM_10_ and PM_2.5_ pollution levels are high in the northwest and low in the southeast. Government can focus on the central region and implement proper measures to strictly control O_3_ emissions while preventing PM pollution in northwestern regions.
(e) The PM_2.5_ concentration in Weifang is negatively correlated with the air temperature and wind speed, while the O_3_ concentration is positively correlated with the air temperature but negatively correlated with the relative humidity and wind speed. This is consistent with the findings that the O_3_ pollution level is high in Summer.

This paper is significant in terms of the in-depth understanding of the spatiotemporal characteristics of air quality and the primary pollutants that affect the AQI in Weifang in recent years. The study provides critical insights into future urban development and pollution control. However, this study has limitations. For instance, the county/district air pollution level is represented by the average of point-monitored concentrations within the area, which may lead to extrapolation errors/bias. Furthermore, air quality is affected by various factors, such as weather and human activities, and there is a lack of quantitative analysis on air quality and other influential factors in this paper. There is a need for future work to combine remote-sensed data and ground-monitored data to control extrapolation errors/bias and to explore relationships between other driving factors and air pollutants for more urban sites.

## Abbreviation

AQI	Air Quality Index
PM_10_	Particles with aerodynamic diameter ≤ 10 µm
PM_2.5_	Particles with aerodynamic diameter ≤ 2.5 µm
NO_2_	Nitrogen Dioxide
SO_2_	Sulfur Dioxide
CO	Carbon Monoxide
AOD	Aerosol Optical Depth
IAQI	Individual Air Quality Index
IAQI_p_	Air quality sub-index for pollutant p
C_p_	Concentration of pollutant p
C_Lo_	Concentration breakpoint that is ≤C
C_Hi_	Concentration breakpoint that is ≥C
IAQI_Lo_	Index breakpoint corresponding to C_Lo_
IAQI_Hi_	Index breakpoint corresponding to C_Hi_
CAAQS	Chinese Ambient Air Quality Standards
